# A Web-Based Interactive Tool to Reduce Childhood Obesity Risk in Urban Minority Youth: Usability Testing Study

**DOI:** 10.2196/formative.9747

**Published:** 2018-11-01

**Authors:** Sandra Verdaguer, Katrina F Mateo, Katarzyna Wyka, Tracy A Dennis-Tiwary, May May Leung

**Affiliations:** 1 School of Urban Public Health Hunter College The City University of New York New York, NY United States; 2 Graduate School of Public Health and Health Policy The City University of New York New York, NY United States; 3 School of Arts and Sciences Hunter College The City University of New York New York, NY United States; 4 The Graduate Center The City University of New York New York, NY United States

**Keywords:** usability testing, interactive technology, mHealth, childhood obesity, minority, health nutrition, health education

## Abstract

**Background:**

Childhood obesity is a serious public health issue among minority youth in the United States. Technology-enhanced approaches can be effective for promoting healthy behavior change.

**Objective:**

The purpose of this study was to test the usability of prototypes of a Web-based interactive tool promoting healthy dietary behaviors to reduce childhood obesity risk in urban minority youth. The Web-based tool comprised a manga-style comic with interactive features (eg, sound effects, clickable pop-ups), tailored messaging, and goal setting, and was optimized for use on tablet devices.

**Methods:**

Latino and black/African American children ages 9 to 13 years were recruited to participate in two rounds of usability testing. A modified think-aloud method was utilized. Self-reported surveys and field notes were collected. Audio recordings and field notes from usability testing sessions were systematically reviewed by extracting and coding user feedback as either positive comments or usability or negative issues. The quantitative data from self-reported questionnaires were analyzed using descriptive statistics.

**Results:**

Twelve children (four female; eight black/African American) with a mean age of 10.92 (SD 1.16) years participated. Testing highlighted overall positive experiences with the Web-based interactive tool, especially related to storyline, sound effects, and color schemes. Specific usability issues were classified into six themes: appearance, content, special effects, storyline, terminology, and navigation. Changes to the Web-based tool after round 1 included adding a navigation guide, making clickable icons more visible, improving graphic designs, and fixing programming errors. In round 2 of testing (after modifications to the Web-based tool were incorporated), many of the usability issues that were identified in round 1 did not emerge.

**Conclusions:**

Results of testing will inform further development and finalization of the tool, which will be tested using a two-group pilot randomized study, with the goal of reducing childhood obesity risk in minority, low-income youth.

## Introduction

### Background

Childhood obesity continues to be a serious public health challenge [1]. In the United States, the prevalence of obesity among youth is 18.5% [2]. The challenge remains pronounced particularly in low-income, minority populations. Latino and black/African American children have the highest rates at 25.8% and 22.0%, respectively [2]. Furthermore, adolescents (12-19 years) have the highest prevalence (20.6%) compared to school-aged (6-11 years; 18.4%) and preschool-aged children (2-5 years; 13.9%) [2]. Childhood obesity leads to negative health outcomes, such as type 2 diabetes, cardiovascular disease, and hypertension, which can continue through to adulthood [3-5]. This complex epidemic has been attributed to, among other behaviors, the increased consumption of energy-dense and low-fiber foods [6,7] as well as the reduced consumption of nutrient-dense fruits and vegetables [8,9].

Effective, yet innovative interventions are needed to capture the attention of children living in a multimedia environment. The pervasiveness of technology and new media use in youth, particularly within the Latino and black/African American population [10-12], highlights opportunities and potential new avenues to engage with this priority population [13]. A systematic review indicated that Web-based programs, as part of a multicomponent intervention, could reduce obesity and overweight in school-aged children [14]. Web-based and technology-assisted interventions, particularly if developed using human-centered approaches and informed by theory [15], have the potential to increase access, improve convenience, decrease cost, and increase participant engagement with dietary behavior change strategies, especially among culturally diverse and hard-to-reach communities [16-19]. At the same time, these types of interventions that allow for flexible engagement with health-related material may require more intrinsic motivation to initiate and maintain engagement over time. Thus, innovative dietary-focused interventions targeting youth should not only incorporate technology, but also integrate engaging features and components to sustain interest and use.

### Theoretical Basis and Content of Intervention INC

Intervention INC is a theory-informed, Web-based interactive tool promoting healthy dietary behaviors, specifically increased fruit and vegetable or water intake, with the goal to reduce childhood obesity risk in Latino and black/African American youth. The main component of the Web-based tool is a novel interactive manga comic, optimized for use on tablet devices. Although research is limited, Japanese comic art, commonly known as manga, has previously been used as part of cognitive behavioral therapy to improve depressive symptoms in Japanese adults [20], as a mental health campaign for youth in England [21], and as an obesity prevention tool for minority children in the United States [22,23]. Unlike Western-style comic books, manga are a unique form of multimodal narrative media that stimulate a reader’s attention by combining detailed visual images and text to create more of a subjective viewpoint of a story [24]. Another distinct feature of manga comics is their wider range of genres. Manga comics are an increasingly popular form of entertainment in many countries, including the United States, irrespective of gender, nationality, or age [25-28]. Although such popularity increases the opportunity for reach of manga comics, components such as story plot and character details (eg, physical features, language use, personal preferences) can be developed to tailor these comics for specific minority populations.

The comic component of the Web-based tool was guided by the narrative transportation theory. The narrative transportation theory explains how narrative communication, such as manga comics, could contribute to changes in health-related beliefs and behaviors by transporting the reader into the narrative world [29]. According to the narrative transportation theory, transportation into a narrative world is believed to lead to acceptance of persuasive messages within a story through multiple mechanisms, which include positive relationships with story characters, lowered resistance to story messages, and similarities to real-world experiences [30-34]. If a reader likes or identifies with a specific character, the events experienced by the character or statements made by the character may have a greater effect in shifting the reader’s beliefs [33,34]. As a result, narrative messages may be more effective than fact-based evidence, particularly when the messages are not similar to one’s own beliefs [29]. Additionally, readers tend to be more engaged with stories that are similar to their personal experiences and cultural values [29]. Thus, embedding health messages into storylines with realistic and relatable scenarios could further engage readers, and thus potentially impact health-related attitudes and beliefs. The narrative transportation theory also suggests that images are most impactful when they are embedded in a story rather than provided in isolation as it could enhance the narrative influence [35]. Therefore, visual images relevant to the story’s message, such as those incorporated in manga comics, may further impact attitudes and beliefs.

Social cognitive theory is a frequently used framework in effective dietary behavior change interventions [36,37], and it also lends explanation to ways in which a manga comic may influence health behavior in youth [22,23]. Exposure to characters in the storylines may facilitate observational learning and influence health behaviors, particularly when readers relate to the comic characters and consider them role models [38]. With input from members of the priority population throughout development, character personalities, interests, and appearances can be designed to increase the likelihood that readers may see them as relatable, and thus role models. The development of similar entertainment-education narratives draws greatly on social cognitive theory by using role models to perform new behaviors [39-41]. Further, the use of relatable characters to illustrate the positive effects of healthy eating and the negative effects of unhealthy eating operationalizes the construct of outcome expectations for comic readers. Thus, an engaging manga comic informed by the narrative transportation theory, which includes health messages and content guided by social cognitive theory, may be an effective vehicle to promote healthy eating behaviors.

Another key component of the Web-based tool is goal setting. Goal setting is discussed in several behavior change theories, including social cognitive theory and goal-setting theory, and involves a commitment to change through small steps [42-44]. These theories similarly relate goals to outcome expectancy and self-efficacy, both of which are needed for goal commitment and attainment. Further, goal setting and self-monitoring are approaches through which self-regulation is operationalized. In the Web-based tool, goal setting, weekly assessment of goals, and tailored messages and feedback (based on initial screening questions and goal assessments) are integrated as theory-guided approaches to support healthy behavior change.

### The Concept of Usability Testing

According to the US Department of Health and Human Services, usability testing refers to evaluating a product or service by testing it with representative users [45]. Usability testing is a crucial step in the development of online health tools and mobile health (mHealth) apps and technologies to ensure that they are accessible, understandable, and useful to end users, and are delivered in an efficient, effective, satisfying, and culturally competent manner [46,47]. Although several studies have emphasized how usability testing can improve technology-based tools [43-45], there is limited research detailing usability testing methods for mHealth tools with youth users, especially younger than 13 years of age [48-53]. A challenge often cited is that traditional usability testing approaches, whether via survey or qualitative methods, are designed for adults and may require different practical, methodological, and ethical considerations with children. The literature also highlights the importance of taking into account individual characteristics that may make it easier or more difficult to participate in these verbal reporting methods, such as level of “extraversion” and “friendliness” [51,53-55]. For example, usability testing done with very young children (younger than 7 years) have highlighted issues related to impatience during testing, unpredictable reactions (especially if the child is uncertain about what to do), and minimal remarks made by users while using a typical think-aloud protocol [55,56]. At the same time, authors have emphasized how behavioral observation (especially during “free play”) often provides the most useful information and insight into usability [55,56]. Although simplifying usability survey questions or think-aloud verbal probes may address issues of literacy and understandability in children, this may also diminish the depth of relevant feedback provided by youth users. Thus, more research is needed to demonstrate successful approaches to usability testing among youth, and particularly among the understudied preadolescent population (9 to 12 years). The lack of published studies in this area suggests that Web-based health promotion tools are being developed without formal involvement or evaluation by potential users, which can impact their potential usefulness, relevance, and effectiveness.

The purpose of this study was to conduct usability testing with Latino and black/African American preadolescents to evaluate prototypes of Intervention INC. Study results will be used to finalize the tool, which will be evaluated in a pilot randomized controlled trial (RCT). This study also aims to add to the limited literature related to usability testing of Web-based tools with youth by describing usability testing methods used to evaluate a Web-based tool with urban minority preadolescents.

## Methods

### Study Overview

This study is part of a larger study that aims to design, develop, and evaluate the Intervention INC tool. Textbox 1 outlines the multiple phases and research activities of the overall study; research activities specific to this study are marked. The methods described focus on the two rounds of usability testing conducted during the development phase with children using prototypes of the Intervention INC tool.

### Sample Participants

English-speaking Latino and black/African American children ages 9 to 13 years were recruited to participate in two rounds of usability testing to provide feedback and identify problems to help inform final development of the Web-based tool. Participants were recruited from a contact list of 36 children, who had participated in previous formative phase focus groups ot interviews (manuscript under review) and early development phase study sessions (manuscript in preparation). These youth were originally recruited via a community-based organization primarily serving children in high-need New York City neighborhoods and local outreach near businesses within the East Harlem, New York, neighborhood.

Eligibility criteria for this prior study sample consisted of the child being between the ages of 9 and 12 years; the child self-identifying as Latino and/or black/African American; the child being English-speaking; the child having internet access, as well as access to a mobile phone or tablet; and the child having an interest in talking about food and technology. We did not screen for reading or digital literacy level as content in the Web-based tool was delivered via multiple mediums, including text, audio, and images. Literature suggests that pictures and audio-assisted reading improves reading comprehension and lowers literacy level of the text [57,58].

Children meeting eligibility criteria were scheduled for a one-on-one usability testing session with a study staff member. Round 1 sessions were conducted in June 2017 and round 2 sessions in July 2017 after certain modifications were made to the Web-based tool. The goal was to recruit five to eight children in each round of usability testing as it has been reported that usability testing with five users will reveal 85% of usability issues [59,60]. Child assent was obtained prior to study participation, in addition to parental permission and a photo release form. Participants received a US $10 gift card and a round trip Metrocard on completion of the session. All study activities were approved by the Institutional Review Board at Hunter College in New York, NY.

Phases and activities of Intervention INC tool design and evaluation. *Research activity specific to this study.

Formative phase

Focus groups or interviews with children and parents

Development phase

- Internal development of initial Web-based tool concepts
- Codesigning of Web-based tool content and design with children and parents
- Usability testing of Web-based tool prototypes with children* and parents

Evaluation phase

Two-group pilot randomized controlled trial to evaluate feasibility and acceptability of Web-based tool with parent-child dyads

**Figure 1:**
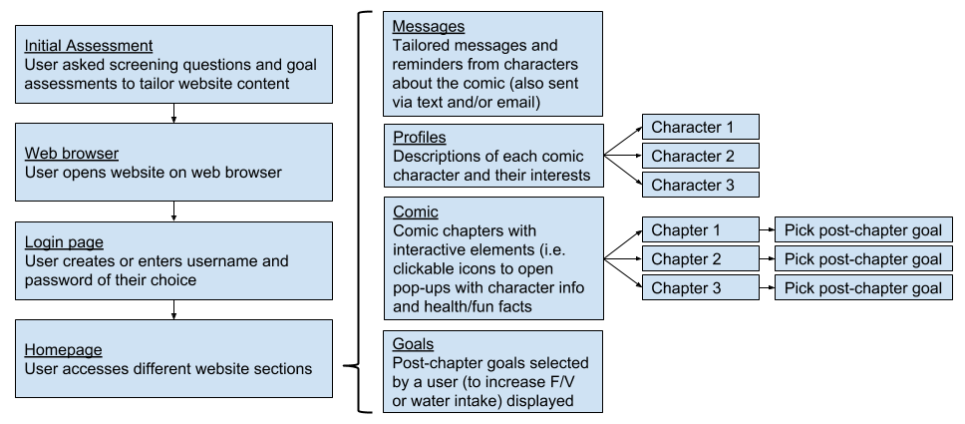
Flowchart of Web-based tool components. F/V: fruit/vegetable.

### Web-Based Tool Description

The Web-based tool tested in round 1 and round 2 consisted of a three-chapter interactive nutrition comic with a character profiles section and embedded interactive features (eg, sound effects, character voice-overs, clickable pop-up windows) to engage users. At the end of each chapter, a tailored message from a character was provided to the user promoting either fruit and vegetable or water intake, as well as a prompt to select a goal related to either increasing fruit and vegetable or water intake (tailored content based on initial screening questions and goal assessments). Figure 1 shows a flowchart of the Web-based tool components that were tested within the current study.

The Web-based tool was optimized for use on tablet devices as formative research with a similar population highlighted that most parents reported owning mobile phones and tablet devices, and a majority of children reported preferring tablets over laptops (manuscript in preparation). For the purposes of comic development, tablets were preferred over mobile phones because of the larger display size and also to maintain the touchscreen capabilities. Throughout development, the tool was tested on devices with iOS, Android, or Windows operating systems and across Safari, Chrome, Firefox, and Internet Explorer Web browsers.

The final version of the Web-based tool will include an additional three chapters (six chapters in total), post-chapter trivia questions, and rewards for correct answers, as well as expanded health information and fun-fact pop-ups throughout each chapter to reinforce health messages. Screening questions will also be incorporated and asked first to determine whether the child receives messages, goals, and comic content focused on either fruit and vegetable or water intake.

### Data Collection

Testing sessions for round 1 and round 2 followed similar procedures. They were conducted in private rooms in a college campus building with two trained researchers (a moderator and a note taker). Demographic information (eg, age, gender, race or ethnicity) and technology use and preferences (eg, “What devices do you use to access the internet or download apps?”) of scheduled child participants were previously assessed during formative phase study sessions via a questionnaire. However, these data were collected from any unscheduled child participants, who attended usability sessions (additional details in Participant Characteristics section). During the usability testing sessions, a combination of qualitative and quantitative methods were implemented. Both methods are essential in the iterative design cycle [61]. Each session consisted of brief think-aloud training, usability testing of the Web-based tool with a modified think-aloud protocol and moderator guide (with examples of prompts to encourage verbalized feedback from participants throughout testing), and a questionnaire to assess usability and acceptability. Each participant accessed the Web-based tool using a touchscreen laptop (Microsoft Surface Pro) as it provided flexibility for the participant to use the device as a computer or tablet. Usability testing sessions were audio-recorded and field notes were taken to document participant’s comments, performance, behaviors, and nonverbal body language.

### Think-Aloud Training

When using think-aloud with adults, examples from the literature suggest starting with a practice session where a moderator or evaluator asks the participant to do an example task similar to the target tasks to orient participants to the practice of talking out loud (as opposed to explaining) before actually engaging with the developed tool or system [62,63]. Once the participant starts interacting with the tool or system, evaluators should only intervene when a participant stops verbalizing their thoughts, and only use simple, short, and nondirective prompts such as “keep on talking” to minimize biasing the user to change their behavior. Prior to the usability testing with our Web-based tool, the moderator explained to participants the purpose of the session (eg, to test an early version of a website using a tablet to get feedback and suggestions on how to make it better) and provided instructions on how to “think aloud” while testing different sections of the website. Our protocol included the moderator explaining the concept (eg, “I want you to say out loud what you are thinking as you use the tablet to go to the website”) and providing an example to practice (eg, “I want you to raise the volume of this tablet while thinking out loud”). This example was practiced until the child demonstrated an understanding of how to “think aloud” (eg, explained out loud that he or she is looking for the volume button on the side of the tablet and pressing the “up” button to raise the volume). In addition, as there is limited literature on using the think-aloud method with youth, moderators were prepared to use more directed prompts and questions in the case that child participants forgot to verbalize their thoughts while using the tool.

### Usability Testing of Web-Based Tool

Participants were first asked if they would prefer to receive a message with a link to the website by text or email. They were then provided with a printed sample text or email message that included the website URL. The moderator asked the participant if he or she knew what to do next (ie, click on website URL or open a browser to type in the URL). Once the website URL was entered into a browser on the tablet, a log-in page was displayed with a form to enter a username and password. The moderator provided the username and password for participants, and observed if the participant was able to enter in the information to log in. Once logged in, participants were allowed to navigate freely through the different sections of the Web-based tool, but were guided to cover all the sections, which included comic chapters, goals, the message board, and character profiles (see Figure 1).

Throughout usability testing of the Web-based tool, participants were encouraged to think aloud to explain what they were thinking as they were navigating through the sections. While reading the comic, the participants were specifically encouraged to read aloud, verbalize reactions, and share initial thoughts with the moderator. Examples of think-aloud prompts included, “What is the first thing you notice on this page?” “Can you tell me what you’re doing?” and “Is there anything you would change?” Prompts were also provided to encourage specific feedback once a child experienced any special effects in the comic or interacted with clickable icons (eg, “What did you think about that animation?” “Why did you click that?” “What do you think of that pop-up message?”).

A note-taking guide was also developed for use by the note taker to record observations of participant’s responses (especially nonverbal) during usability testing of the comic section. The note-taking guide included screenshots of each panel of the comic, along with multiple checkboxes (eg, to check which automatic animations displayed automatically), yes or no options (eg, to indicate if user selected a goal), and reminders for the note taker to record start and end times and note general comments. Using this guide, data related to time taken to complete each comic chapter, number of usability issues, frequency of interaction with clickable features within the comic, and specific comments made in each panel of the comic could be collected.

### Perceived Usability and Acceptability

The perceived usability and acceptability of the Web-based tool was assessed using a questionnaire provided to each participant after the usability testing session. The questionnaire was administered via pen and paper, and the moderator was available to answer any questions about the survey or clarify words that the participant did not understand. The moderator additionally highlighted that this questionnaire was not meant to test the child but was a way for the child to express how easy or hard it was to use the Web-based tool so that the developers could improve it for future users.

The questionnaire combined and modified items from the System Usability Scale (SUS) [64], the Usefulness, Satisfaction, and Ease of use (USE) questionnaire [65], and an acceptability/usability measure questionnaire [66] in order to assess five usability domains: usability, usefulness, ease of use, ease of learning, and satisfaction. The combined questionnaire consisted of 37 items scored on a five-point Likert scale from strongly disagree to strongly agree. The usability domain comprised the 10 items from the SUS questionnaire. Two items comprised the usefulness domain (one from the USE and the other from the acceptability/usability measure). Ease of use domain was assessed using 13 items (10 from the USE and three from the acceptability/usability measure). Ease of learning was assessed by the same four items found in the USE questionnaire. The satisfaction domain comprised eight items (all from the USE, except for one that was added from the acceptability/usability measure).

The combined questionnaire was pilot tested in earlier development phase study sessions with a similar population of children ages 9 to 12 years (manuscript in preparation). Based on this previous testing, some modifications were made to tailor the questionnaire according to children’s literacy levels for this study. For example, the item “I found the system very cumbersome to use” was replaced with “I found the website very awkward to use,” and “I would imagine most people would learn to use this system very quickly” was changed to “I think most people my age would learn to use this website very quickly.” Additionally, changes were made to make the questions more appropriate for our Web-based tool. For example, the word “system” or “tool” was replaced with the term “website.”

### Data Analysis

Analysis of think-aloud data, including coding categories and themes were guided by approaches used in previous literature [46,67,68]. Audio recordings from usability testing sessions were not transcribed verbatim as the context of user interactions with the tool (eg, audio of character dialog prompted by touching interactive icons) would be more evident from listening to and directly analyzing audio recordings [69,70]. Microsoft Excel version 15.33 was used to assist with data organization and analysis. For both round 1 and round 2, the audio recordings and field notes were systematically reviewed. First, child utterances during usability testing were extracted and coded as either positive comments or usability issues (which also included negative comments verbalized by participants). Similar or related comments were then grouped into themes and subthemes. Each code was counted in coding units. Coding units consisted of sentences or reactions from the participants and programming glitches counted during usability testing. The major coding rules were as follows: (1) multiple sentences or reactions that referred to the same matter were coded as one unit (eg, if a participant made multiple comments about a picture being too small, they were all counted as one unit); (2) agreements between participants on the same matter in dyad sessions were counted as two units (eg, if a participant made a comment and his or her pair agreed, the two comments were counted separately); and (3) programming glitches that occurred during dyad sessions were counted as one unit.

To ensure the reliability of the content analysis, the coding and themes were continually validated by two other researchers throughout the analysis process. Specifically, the primary analyst coded the data and then presented the analysis to two other researchers, who reviewed code application to comments or verbalizations. If any inquiries or disagreements arose regarding codes and themes, the three researchers discussed and resolved any discrepancies. Coding revision and theme refinement continued until data analysis was complete. Field notes were reviewed to help inform analysis.

The quantitative data from self-reported questionnaires about participant’s usability and acceptability of the Web-based tool across the five domains were analyzed using SPSS version 22 and Microsoft Excel version 15.33 to calculate the means, standard deviation, and ranges (minimum-maximum) for the overall score as well as subscales. For usability domain questions (10 items), separate means, standard deviation, and ranges were also calculated based on the SUS scoring protocol [64].

## Results

### Participant Characteristics

A total of 12 children (n=6 per round) were recruited. Round 1 consisted of two dyad sessions and two individual sessions and round 2 consisted of six individual sessions. Although dyad sessions were not a part of the initial study design, they were conducted in round 1 as two scheduled children brought their relatives. The overall age of participants was mean 10.92 (SD 1.16) years (range 9 to 13 years). The mean age of participants in round 1 was slightly higher than in round 2 (mean 11.17, SD 1.33 years and mean 10.67, SD 1.03 years, respectively). The majority of participants were male (n=8) and black/African American (n=8). Among the 12 participants, eight (three in round 1; five in round 2) were involved in the codesigning process of the Web-based tool and participated in a previous usability session of the first prototype.

### Technology Use

Prior to accessing the Web-based tool, participants were asked whether they would prefer to receive messages about the Web-based tool through text message or email. Participants’ preference was text message (7/12, 58%) over email (5/12, 42%). The most common devices used to access the internet or download apps were tablets (11/12, 92%) and mobile phones (10/12, 83%), followed by desktop computer or laptop (8/12, 67%) and xBox (5/12, 42%). Other devices participants reported to use to access the internet or download apps were iPod, Wii, and Kindle. Mobile phones and tablets were the top two devices used most often. However, mobile phones were the preferred devices among participants (8/12, 67%). Although the majority of participants used mobile phones, two of 12 indicated they did not use mobile phones. Among the participants who used mobile phones, four of 10 shared their mobile phones with someone else in the family, normally with their mom and siblings. Table 1 summarizes the participants’ demographic and technology characteristics.

### Usability Testing Themes

Overall mean testing time was 65 (SD 12) minutes with mean time in round 1 slightly higher than in round 2 (mean 67, SD 8 minutes vs mean 63, SD 15 minutes, respectively). Testing revealed a total of 586 comments or reactions. A greater number of comments and reactions were collected in round 1, especially during dyad usability sessions (329 collected in round 1, 257 collected in round 2). Multimedia Appendix 1 provides a summary of participants’ comments and reactions identified from the content analysis, which have been classified under six themes: appearance, content, special effects, storyline, terminology, and navigation. Additional comments were labeled under general feedback. Participants’ comments and reactions were further categorized as either positive comments or usability issues. Overall, there were more positive comments (70.8%, 233/329 in round 1; 65.8%, 169/257 in round 2) compared to usability issues (29.2%, 96/329 in round 1; 34.2%, 88/257 in round 2) in both rounds.

**Table 1:** Demographic characteristics and technology use of participants.

Characteristics		Round 1 (n=6)	Round 2 (n=6)	Total (N=12)
Age (years), mean (SD)		11.17 (1.33)	10.67 (1.03)	10.92 (1.16)
**Gender, n**				
	Male	4	4	8
	Female	2	2	4
**Race, n**				
	Black/African American	4	4	8
	Latino	2	2	4
**Preferred notification platform, n**				
	Text message	4	3	7
	Email	2	3	5
**Devices used to access internet or download apps, n**				
	Tablet	6	5	11
	Mobile phone	5	5	10
	Desktop computer or laptop	3	5	8
	Xbox	3	2	5
	Other (eg, iPod, Wii, Kindle)	2	1	3
**Type of smartphone, n**				
	Android (eg, Samsung)	4	2	6
	iPhone	1	3	4
	Do not use a phone	1	1	2
Participants who share mobile phone with other family members, n		3	1	4
Participants who have been involved in the codesigning process of the Web-based tool, n		2	3	5
Participants who have been involved in a previous usability session, n		1	2	3

Appearance referred to the impressions of how the Web-based tool looked and included the design, layout, illustrations, font, and colors. Participants approved of the comic illustrations and the overall design of the Web-based tool. One participant mentioned this referring to the illustrations of the comic: “I like it’s [the comic] anime.” However, they had complaints on the colors as the comic was in black and white with only some instances of color. One participant expressed “I would like it [the comic illustrations] better in color, we are in 2017!”

Content included information that was delivered through the Web-based tool. Participants found the information provided in the character profiles section most interesting. Participants expressed particular interest in the character’s favorite recipes, hobbies, and favorite links (ie, external online games and apps). One participant mentioned wanting to know the character’s favorite color. Participants also reported liking the fun facts. For example, one participant said “Interesting, I didn’t know that [basketball fun fact].” However, three round 2 participants felt that some of the pop-ups and post-chapter messages were “off topic” or not relevant to the story. Participants in round 1 suggested that a guide could be added to learn how to use the interactive features within the comic. However, after the guide was included, some round 2 participants commented that although they thought the guide was useful, it was not necessary to include it at the start of each chapter.

Special effects were comic features, including sound effects, voice-overs of some selected character dialog, clickable pop-up windows with additional information, and animation, meant to increase immersion into and engagement with the comic. Participants commented positively on them and asked for more special effects. Suggestions were even provided as to specific scenes in the comic where additional special effects could be incorporated. Some of the quotes were: “It is funny that he’s [the chameleon] blinking his eyes” and “It would be cool if they [the characters] were moving. Kind of funny too.”

Storyline comprised any comment related to the plot of the nutrition comic. Overall, the storyline was positively received, especially the flashbacks (ie, of character memories) and the “love triangle” between characters. Participants were generally very engaged while reading the comic, often using vocal inflections to express reactions or, at times, reading character dialog out loud and mimicking the character voice. In general, participants thought the comic was humorous and chapters had interesting endings, which made them eager to read subsequent chapters. Participants also mentioned liking the characters and relating to at least one of them. However, there were parts of the storyline where the older participants had other expectations. One participant mentioned, “That’s it? The worm thing...Oh, I thought it would be something different.”

Terminology referred to the words, abbreviations, and onomatopoeia used in the Web-based tool. There were a few words that participants had trouble reading, such as “high-fructose corn syrup” and “hypertonic solution.” Participants stated they did not know the meaning of some words and abbreviations (“What does NPS mean?” “What is an athlete?”). No problems were encountered with the onomatopoeia as children correctly identified the intended sounds.

Navigation reflected the way a user navigated the Web-based tool to complete tasks. For one participant, the steps that should have been followed to access the Web-based tool (ie, open a browser and typing in URL) were unclear. Three participants also pointed out that they did not know what to do after completing a section or a task. Their suggestions included adding some guidance texts such as “type this link into your browser” and “check back next week for a new chapter.” On the other hand, participants also provided positive comments related to navigation. Turning pages was often an issue for participants as the touch area to “swipe” was narrow and not as obvious to users. Four participants were confused on how to go back to the main page, commenting that “you should make Home link bigger and more obvious.” One participant said, “I love being able to swipe and zoom in.”

General feedback included any other broad comments related to the Web-based tool. Overall, participants’ general feedback was very positive. For example, two participants said, “I liked it [the Web-based tool], I want it on my phone!” and “I would give it [the Web-based tool] a 9.9!!!”

### Modifications to the Web-Based Tool Between Rounds 1 and 2

Although round 1 participants provided multiple suggestions and different usability issues were detected, modifications to the Web-based tool between round 1 and round 2 had to be prioritized. Prioritization adjustments were based on what the researchers believed would have the largest positive effect on usability. Additionally, adjustments were chosen based on the time, resources, and skills available on the development team.

**Figure 2:**
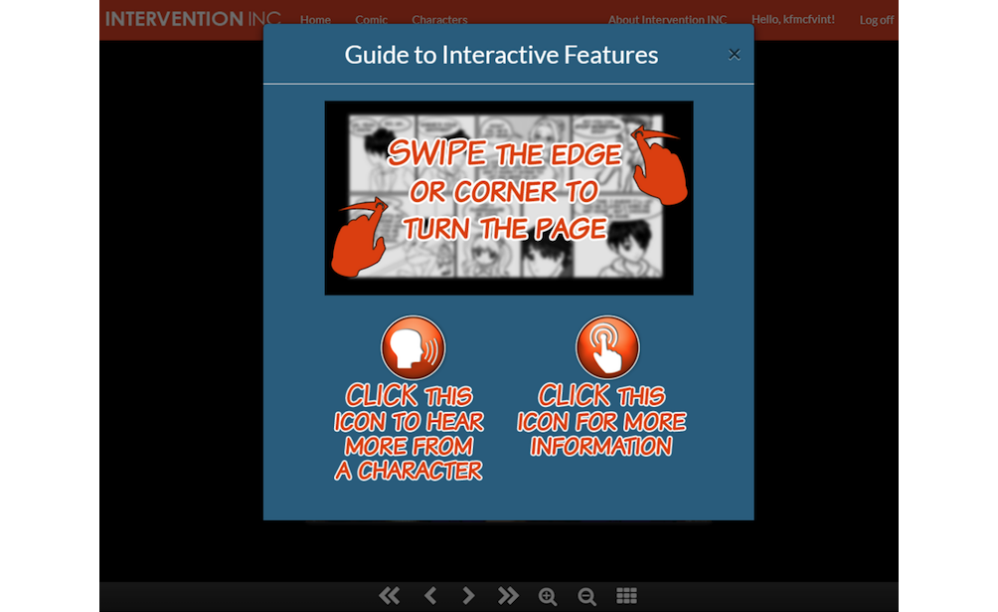
Screenshot of the navigation guide added as a modification to the Web-based tool after round 1 of usability testing.

**Figure 3:**
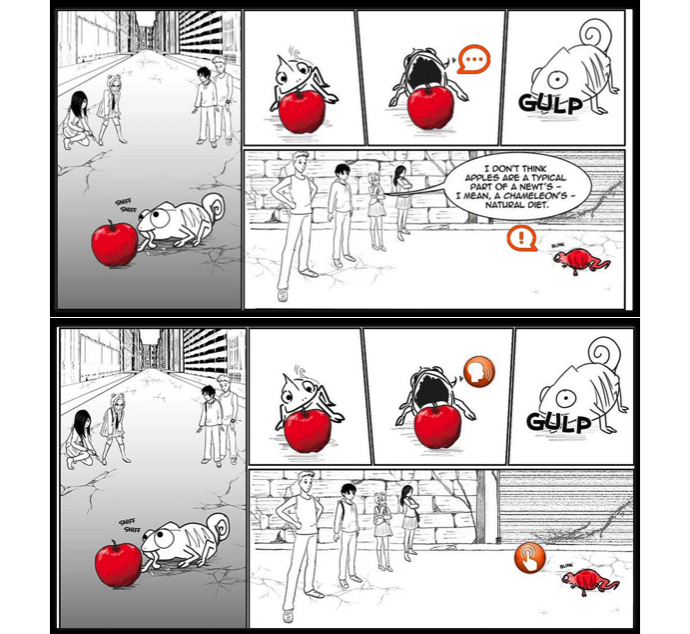
Screenshots of the clickable icon modification added to the Web-based tool after round 1 of usability testing premodification (top) and postmodification (bottom).

**Figure 4:**
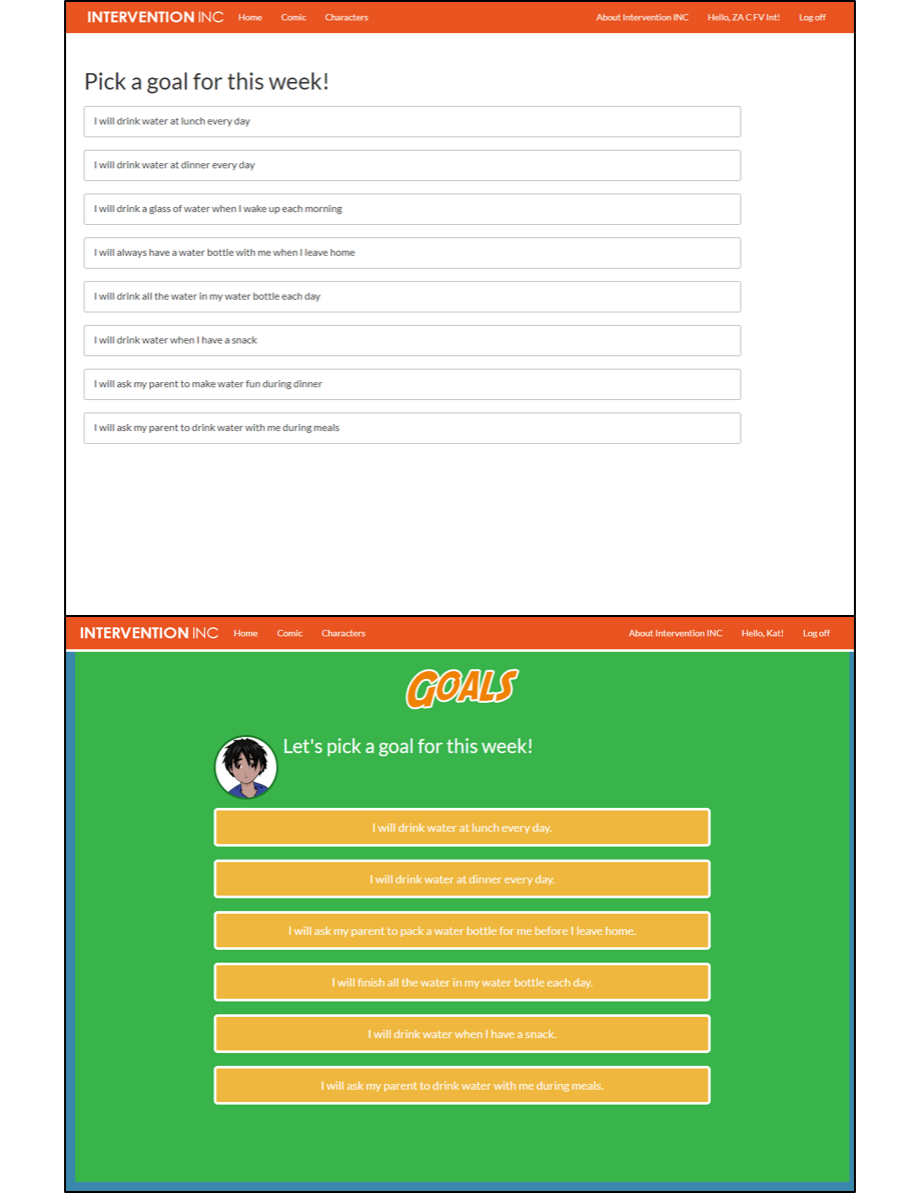
Screenshots of the goal setting modification added to the Web-based tool after round 1 of usability testing premodification (top) and postmodification (bottom).

The issues and problems highlighted by round 1 users that we sought to address with modifications between round 1 and round 2 included the following: (1) clickable icons for information pop-ups, sound effects, or character dialog were not obvious; (2) the touch feature to “swipe” pages was not intuitive; (3) the siblings in the comic story did not look related; and (4) multiple programming errors were identified (eg, tips not displaying after goals being selected, sound effect of “swiping” page not playing). Modifications to the Web-based tool to address these issues after round 1 included (1) making clickable icons more obvious and visible (changing shape, color, and pop-out effect), and improving graphic design, such as a making a more unified and vibrant color scheme, forms; (2) adding background and pop-up images; (3) adding a navigation guide to highlight how to identify and use touch features, including clickable icons and “swiping” the comic pages; (4) altering or improving comic illustrations; and (5) fixing programming errors. Figures 2-4 are screenshots of some of the additions and modifications to the Web-based tool.

The modifications may have impacted users’ usability in round 2. Although none of the six round 1 participants clicked the special effects icons initially without being prompted by the moderator, all six round 2 participants selected these clickable icons without any prompts. Also, the proportion of participants who navigated the comic pages by swiping was higher in round 1 (from 1/6 in round 1 to 3/6 in round 2). Additionally, it may have been clearer to round 2 users that they needed to select a goal at the end of each comic chapter (see Figure 4). In round 1, only 2 of 6 participants understood that they had to select a goal after viewing the goal-setting page for the first time at the end of the chapter. However, all six round 2 participants selected a goal without prompting by the moderator. Lastly, there was an 80% reduction in unique programing glitches and errors in round 2 after modifications to the Web-based tool were made after round 1 (20 reported in round 1, 4 reported in round 2).

**Table 2:** Participant’s perceived usability and acceptability of the Web-based tool.^a^

Domain (37 items)	Round 1 (n=6)		Round 2 (n=6)		Combined (n=12)	
	Mean (SD)	Range	Mean (SD)	Range	Mean (SD)	Range
Usability (10 items)	4.40 (1.04)	3.80-4.80	3.80 (1.16)	3.10-4.60	4.10 (1.14)	3.10-4.80
Usefulness (2 items)	4.67 (0.65)	3.50-5.00	3.67 (1.23)	3.00-5.00	4.17 (1.09)	3.00-5.00
Ease of use (13 items)	4.50 (0.85)	4.31-4.69	4.00 (1.04)	2.85-4.92	4.25 (0.98)	2.85-4.92
Ease of learning (4 items)	4.96 (0.20)	4.75-5.00	4.25 (0.85)	3.25-5.00	4.60 (0.71)	3.25-5.00
Satisfaction (8 items)	4.73 (0.49)	4.38-5.00	4.23 (1.02)	2.88-5.00	4.48 (0.83)	2.88-5.00
Total	4.58 (0.81)	4.41-4.76	4.00 (1.07)	3.19-4.81	4.29 (0.99)	3.19-4.81

^a^Assessment questionnaire was developed by using a combination of items from the System Usability Scale [64], Usefulness, Satisfaction, and Ease of use questionnaire [65], and acceptability/usability measure [66]. Response options ranged from 1 (strongly disagree) to 5 (strongly agree).

### Participant’s Usability and Acceptability Questionnaire

Table 2 highlights the mean scores of the five usability domains (usability, usefulness, ease of use, ease of learning, and satisfaction) for round 1, round 2, and combined (rounds 1 and 2). The combined total score of perceived usability and acceptability of the Web-based tool was high (total mean 4.29, SD 0.99, range 3.19-4.81). Additionally, all five usability domains had combined scores of over 4.00. Specifically, the ease of learning and satisfaction domains had the highest combined scores (mean 4.60, SD 0.71, range 3.25-5.00 and mean 4.48, SD 0.83, range 2.88-5.00, respectively). In round 1 specifically, all domains had a mean score higher than 4.00, ranging from mean 4.40 (SD 1.04, range 3.80-4.80) for usability to mean 4.96 (SD 0.20, range 4.75-5.00) for ease of learning. In round 2, three out of five domains had a mean score of 4.00 or greater. The usefulness and usability domains scored lowest with scores of mean 3.67 (SD 1.23, range 3.00-5.00) to mean 3.80 (SD 1.16, range 3.10-4.60), respectively. Only two individual questionnaire items of the 37 had a mean score lower than 3.00. In round 1, the item “I can use it without written instructions” (item under ease of use domain) had a mean score of 2.67 (SD 1.03, range 1.00-4.00). However, the same item in round 2 had a mean score of 4.00 (SD 1.10, range 2.00-5.00). In round 2, the item “I felt very confident using the website” (item under usability domain) had a mean of 2.33 (SD 1.51, range 1.00-5.00). This same item in round 1 had a mean score of 4.67 (SD 0.52, range 4.00-5.00). In separate scoring of the usability domain questions (10 items) according to the SUS protocol [64], the overall usability was relatively high (total mean 77.08, SD 13.97), with round 1 participants rating the usability of the Web-based tool higher than round 2 participants (mean 85.00, SD 8.94 and mean 69.17, SD 14.11, respectively).

## Discussion

### Principal Findings

This study describes the methods and results of usability testing of Intervention INC, a Web-based tool to promote healthy dietary behaviors in Latino and black/African American youth. Overall evaluation of the prototypes tested over two rounds revealed positive experiences with the Web-based interactive tool and opportunities to incorporate additions to increase engagement and improve usability.

We observed that round 1 participants did not engage with interactive clickable icons. Further probing revealed that, in most cases, users overlooked these icons despite moderators noting that there were interactive features in the comic. Adding a “Guide to Interactive Features” at the beginning of each chapter may have addressed this usability issue, evident by the fact that all round 2 participants clicked on the icons without prompting by the moderator. Except for incorporating a guide, no other content was added to the Web-based tool. However, three round 2 participants mentioned that certain pop-ups and post-chapter messages were “off topic” or not relevant. As each comment made by these participants was counted as a usability issue, this may have contributed to a higher number of content issues noted in round 2. Also, some participants were not familiar with browsers and one participant experienced difficulties when asked to type in URL links. Usability testing revealed the importance of providing training or including user guides for technology-based tools. This is consistent with a previous study in which youth participants needed a short training session prior to engaging with a Web-based program which was focused on increasing physical activity [71]. Although youth are familiar with technology and tablet-optimized tools such as apps, they may need some training at the beginning of Web-based interventions to learn how to access online tools.

Usability testing also reaffirmed the feasibility and acceptability of embedding health information into narratives, as well as the importance of using interactive features to enhance engagement and assist with accessibility. For example, interactive features such as pop-ups with specific health information and accompanying images may increase engagement with the content. In addition, the use of embedded (clickable) audio recordings for long character dialog can help with the literacy of the comic [72]. Overall, the use of a comic-style narrative to communicate health information is an approach to delivering content to low-literate readers [73,74]. In our study, all participants demonstrated great interest in the comic storyline and interactive features (ie, special effects, interactive pop-up, and swiping pages), and in some cases even provided suggestions on how to increase interactivity with the tool. This finding supports other usability studies conducted by the Nielsen Norman Group (leading user interface and user experience consulting firm), which concluded youth younger than age 12 years prefer animation and sound effects and enjoy “hunting for things to click” [75].

From the usability issues identified during round 1, modifications were made, such as incorporating an interactive feature guide and improving the comic’s graphic designs (eg, improved clickable icons and character features). These modifications appeared to enhance round 2 usability based on observation and qualitative feedback. In addition, the improved score for the questionnaire item “I can use it without written instructions” from round 1 to round 2 may be a positive indicator of the impact of an incorporated user guide. However, in general, scores from the usability and acceptability questionnaire (both from the overall questionnaire and just the SUS usability questionnaire items) were slightly higher in round 1 than in round 2. Although the sample was not large enough to make powered comparisons, the scores may have dropped because round 2 had a higher number of participants who were involved in the codesigning process or a previous usability session. Those who participated in previous Web-based tool development activities may have had higher expectations of the Web-based tool than the participants who did not have prior exposure. Age could be another explanation as children become substantially more Web-savvy as they get older [76,77]. Round 1 participants were, on average, half a year older than round 2 participants. Half a year may be a significant amount of time in relation to cognitive or literacy development, particularly with school-aged youth [78]. This age difference (and possible differences in reading or computer and digital literacy associated with age) could also explain why round 1 participants scored the item “I felt very confident using the website” with a much higher mark than round 2 participants. In addition, since some round 1 participants interacted with the tool as a dyad, they may have perceived the tool as having higher usability because they were able to navigate through the tool with a partner. Even if a child may have encountered a usability issue, these may not have been captured or explicitly experienced if the other child was not experiencing the same issue or helped the other child either consciously or unconsciously.

### Modified Think-Aloud Approach Used With Our Participants

Previous studies recommend conducting usability testing with potential users prior to outcome assessment in studies involving larger samples [48,79,80]. The think-aloud method is commonly used as a usability testing approach among adults [81-83]. However, there are limited references in the literature describing the think-aloud approach in youth usability testing, and most have been conducted with older youth [46,84]. For this study, we modified this method by helping youth to express what they were thinking with directed questions and probes. We found that using a modified think-aloud approach was successful in eliciting important feedback to improve user experience. Usability guidelines recommend limiting testing sessions with youth to less than 25 minutes or using multiple stations to break up and vary the modes of engagement [85]. However, we were able to successfully keep youth engaged in usability testing for more than 60 minutes. Our approach provided structured and continuous opportunities for participants to verbalize their thoughts and encourage feedback. In addition, although participants were not asked to read aloud, most of the kids preferred to. This allowed us to successfully identify reading and comprehension issues, which were addressed in the final Web-based tool.

It should be noted that our protocol aimed to conduct individual sessions. However, two dyad sessions were conducted in round 1, and we observed a greater number of comments in round 1 compared to round 2. One explanation for this is that having two participants in round 1 sessions provided many more comments than the individual sessions. Future usability testing of Web-based tools with youth using a modified think-aloud approach should consider dyad assessments (rather than individual) to facilitate more meaningful feedback in a peer-to-peer environment. Indeed, some of the limited evidence of usability testing with youth have discussed the benefits of a similar approach, referred to as “constructive interaction,” and the impact of different factors (eg, nonacquainted vs acquainted dyads, same gender dyads) on the identification of usability problems [86-89].

### Implications for Future Research

Our study highlights the need for further research to be conducted to refine the approaches utilized and to further elaborate on our initial findings related to usability testing with youth, particularly with minority, urban preadolescents. However, multiple insights were gained during this study. First, the modified think-aloud approach used with preadolescents, especially in dyad sessions, were successful in collecting meaningful feedback. In future usability studies, we would continue to engage dyads, in combination with individuals, to evaluate Web-based tools with youth. Secondly, although we encouraged the participants to read the comic aloud, this was not mandatory. During future testing, we would request that all participants read aloud as this would allow for the proper assessment of literacy levels and identification of any reading and comprehension issues across all participants. From an evaluation perspective, we were unable to make direct comparisons between round 1 and round 2 as the participants differed between these two rounds of testing. Thus, future research may consider using the same participants across usability testing rounds, such that direct assessments and comparisons could be conducted. This approach may also have the added benefit of the ability to assess the impact of added or changed components (such as a navigation guide) on usability. Lastly, including a third round of usability testing to study how participants interact with the final product in a real-world setting (without guidance of a moderator) would have been informative. During this third round of usability testing, uninterrupted observational approaches such as screen recordings to capture interactions, along with participants’ voice (audio) as they are completing key tasks, would provide insight into any usability issues that may be encountered outside of a testing setting.

### Limitations

It is acknowledged that this study is not without its limitations. First, the data analysis was performed by one researcher. However, the coding process was continually validated by two other researchers. Secondly, some participants had previously participated in the initial development process of the Web-based tool or a previous usability testing session. This may have contributed to biases regarding certain preconceived ideas for how the Web-based tool would look like or how the storyline was actualized in the comic. However, engaging the same participants throughout tool and intervention development builds on prior knowledge and exposure to the tool, which may contribute to more relevant and informed feedback regarding needed improvements and criticisms [90].

In addition, although the usability questionnaire used in this study was informed by several usability questionnaires commonly used in the literature [64-66], the final combined version is not a validated tool and was only pilot tested in previous development phase study sessions. The general high usability ratings among users and the lack of difference between round 1 and round 2 scores on the self-reported questionnaires for participant’s usability and acceptability of the Web-based tool are also suggestive of response bias, which has been observed in other studies using usability questionnaires with youth [91]. Furthermore, although there is always a risk of social acceptability bias while administering surveys with a moderator present, which may be higher with youth [91,92], it was important to ensure that a study staff member was available to clarify terminology or address questions, especially as children have varying levels of literacy. Lastly, one of the usability sessions in round 2 was not recorded due to technical issues. Although field notes were taken during this session, some comments and reactions may not have been documented.

### Conclusions

Usability testing is critical during the developmental process of Web-based tools because it can enhance a tool’s usefulness, engagement, and potential effectiveness for end users. This study adds to the limited literature related to usability testing of Web-based tools with youth by describing modified usability testing methods used to evaluate the Intervention INC tool with urban minority preadolescents. The authors engaged youth during usability testing sessions using a combination of a modified think-aloud approach with directed questions and prompts, behavioral observation of users interacting with the tool, and a usability questionnaire. Usability findings suggest that this Web-based tool was acceptable to youth and could be an engaging approach to communicate and promote healthy dietary behaviors among urban minority youth.

Results from this study will inform further development and finalization of the Web-based tool, which will be tested using a two-group pilot RCT targeting fruit and vegetable or water intake to reduce childhood obesity risk in black/African American and Latino youth. The final tool will be a six-chapter comic with one chapter being released each week. If such a tool is found to be effective in larger scale studies, it could be disseminated as a publicly available online health promotion tool that could be implemented in various settings, such as health care clinics, after school-based programs, and public schools, which highlights its potential for high reach.

## Abbreviations

RCT: randomized controlled trial
SUS: System Usability Scale
USE: Usefulness, Satisfaction, and Ease of use questionnaire

## Appendix

Multimedia Appendix 1 Themes identified from a content analysis of usability testing data.
