# Supplementary material for: A Web-Based Interactive Tool to Reduce Childhood Obesity Risk in Urban Minority Youth: Usability Testing Study
Source: JMIR Form Res. 2018 Nov 1;2(2):e21. doi: 10.2196/formative.9747 (PMC6334715; doi:10.2196/formative.9747)
Supplement: Multimedia Appendix 1 [file formative_v2i2e21_app1.docx]

| Themes and subthemes | | Frequency of units | |
| --- | --- | --- | --- |
|  | | Round 1 | Round 2 |
| **Appearance** | |  |  |
| **Total positive comments** | | **44** | **41** |
|  | Liked drawings | 25 | 22 |
|  | Liked the design | 9 | 13 |
|  | Liked the colors | 10 | 6 |
| **Total usability issues** | | **23** | **15** |
|  | Drawing issues | 4 | 7 |
|  | Design issues | 15 | 6 |
|  | Color issues | 4 | 2 |
| **Content** | |  |  |
| **Total positive comments** | | **41** | **33** |
|  | Liked the info given | 33 | 31 |
|  | Easy and simple | 8 | 2 |
| **Total usability issues** | | **7** | **24** |
|  | Add content | 5 | 7 |
|  | Change content | 2 | 3 |
|  | Confusing | 0 | 5 |
|  | Off topic | 0 | 6 |
|  | Delete | 0 | 3 |
| **Special effects** | |  |  |
| **Total positive comments** | | **49** | **37** |
|  | Liked the sound effects | 19 | 4 |
|  | Liked the dialog | 14 | 18 |
|  | Liked animation | 16 | 15 |
| **Total usability issues** | | **14** | **8** |
|  | Suggestion | 13 | 6 |
|  | Dislike | 1 | 2 |
| **Storyline** | |  |  |
| **Total positive comments** | | **85** | **48** |
|  | Found humor | 50 | 19 |
|  | Liked storyline | 16 | 20 |
|  | Relatable | 6 | 4 |
|  | Other positive reactions verbal and nonverbal | 13 | 5 |
| **Total usability issues** | | **21** | **13** |
|  | Disconnect to expectation | 8 | 3 |
|  | Suggestion/dislike | 9 | 6 |
|  | Unrelatable | 1 | 0 |
|  | Too long | 1 | 3 |
|  | Unclear | 2 | 1 |
| **Terminology** | |  |  |
| **Total usability issues** | | **14** | **8** |
|  | Confusing | 8 | 6 |
|  | Difficult | 6 | 2 |
| **Navigation** | |  |  |
| **Total positive comments** | | **3** | **0** |
|  | Liked operation features | 3 | 0 |
| **Total usability issues** | | **17** | **20** |
|  | Unclear | 13 | 10 |
|  | Difficult | 1 | 4 |
|  | Failed path | 3 | 6 |
| **General feedback** | |  |  |
|  | Positive comments | 11 | 10 |
|  | Negative comments | 0 | 0 |
